# Supplementary material for: Characterizing cell-type spatial relationships across length scales in spatially resolved omics data
Source: Nat Commun. 2025 Jan 3;16:350. doi: 10.1038/s41467-024-55700-1 (PMC11699133; doi:10.1038/s41467-024-55700-1)
Supplement: Supplementary file 3 — Supplementary Software 1 [file 41467_2024_55700_MOESM3_ESM.zip › CRAWDAD/plots/spleen/3_spleen_pkhl_grid_66_77_tissue_zoom.pdf]

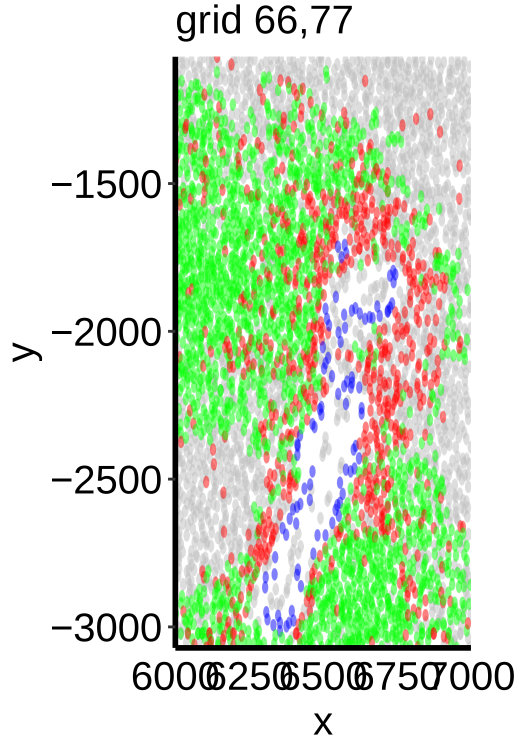

grid 66,77
y
−1500
−2000
−2500
−3000
6000
6250
6500
6750
7000
x
